# Iris juvenile xanthogranuloma in an infant - spontaneous hyphema and secondary glaucoma

**DOI:** 10.22336/rjo.2017.41

**Published:** 2017

**Authors:** Anca Pantalon, Tudor Ștefănache, Mihai Danciu, Sabina Zurac, Dorin Chiseliță

**Affiliations:** * Opthalmology Clinic, “Sf. Spiridon” University Hospital, Iași, Romania; ** Ophthalmology Unit, “Gr. T. Popa” University of Medicine and Pharmacy, Iași, Romania; *** Department of Pathology, “Gr. T. Popa” University of Medicine and Pharmacy, Iași, Romania; **** Department of Pathology, Faculty of Dental Medicine, “Carol Davila” University of Medicine and Pharmacy, Bucharest, Romania

**Keywords:** juvenile xanthogranuloma, secondary glaucoma, infant, spontaneous hyphema

## Abstract

Juvenile xanthogranuloma (JXG) is a benign histiocytic skin disorder mainly encountered during infancy and childhood. Although with multiple potential localizations, less than 1% of the cases exhibit ocular manifestations. Some of these might lead to serious complications, specifically, secondary glaucoma that can result in severe and blinding eye disease.

The aim of the present case report was to demonstrate typical clinical features, emphasize the difficulties attributed when managing these patients and literature review. We present the case of 4 months old female baby with spontaneous hyphema and secondary unilateral glaucoma due to ocular JXG.

The natural history and treatment of the condition were extremely difficult to handle due to multiple opinions in histopathology related to other severe conditions that resembled with the lesions detected in this case: myelomonocytic leukemia and Langerhans cell histiocytosis. Although a minority of patients with JXG have ocular involvement, recognition of this condition is important because a treatment delay can lead to serious complications, such as glaucoma and spontaneous hyphema, as in our case. A thorough differential diagnosis represents the key to a proper management plan in these patients, both on short and long term. “Triple disease” defined as JXG plus neurofibromatosis type 1 (NF-1) and juvenile chronic myelogenous leukemia (JCML) has been reported, but it was not confirmed in our patient.

## Introduction

Juvenile xanthogranuloma (JXG) is a benign non-Langerhans histiocytic skin disorder mainly affecting infants and children, 75% of the cases occurring in the first 9 months of life [**1**]. In 5–17% of the cases, the skin lesions may appear soon after birth [**2**]. About 10% of the cases can manifest in adulthood, being known as “adult xanthogranuloma” [**1**]. The etiology is unknown, although it may represent a reactive process (physical or infectious) [**3**,**4**]. Cutaneous lesions are asymptomatic and generally appear spontaneously during the first year of life as reddish-yellow nodules, most often in the head and neck region. The general tendency is towards spontaneous resolution until the age of 3-6 years old [**3**]. Extra-cutaneous JXG most commonly affects the eye but can involve the brain, lungs, liver, spleen, and other sites. Eye involvement (iris, choroid, retina, optic nerve, and orbit) has been reported to occur in approximately 0.3% to 10% of the children with cutaneous JXG [**5**]. Solitary lesions are found in 60–82% of the cases [**1**]. Among ocular sites, iris has been most commonly involved; some cases might lead to serious complications, from spontaneous intraocular hemorrhages, secondary glaucoma to blinding eye disease. 

Contrary to other xanthomatous diseases, JXG has not been associated with lipid disturbances or metabolic disorders [**6**], but a variety of systemic diseases, including neurofibromatosis type 1 and juvenile chronic myelogenous leukemia, which may be associated with juvenile xanthogranuloma. Patients with this combination of findings require special management.

## Case report

We present the case of a 4 months old female baby referred to our ophthalmology unit by a fellow colleague for unilateral red eye, photophobia, epiphora, and spontaneous hyphema (OD). As a personal history, soon after birth (at 2 weeks), the mother recalled the appearance of few red-orange cutaneous nodules (thorax, head, neck); in time, lesions became more numerous and some changed aspect due to crust formation or mild superficial hemorrhages. As such, a dermatologist was consulted and skin biopsies from the lesions were indicated. The diagnosis established by several pathologists varied greatly, from Langerhans cells histiocytosis to myelomonocytic leukemia or juvenile xanthogranuloma (JXG). Due to this situation, the baby underwent a bone marrow biopsy that excluded any malignant process and pleaded for cutaneous JXG. At this stage, no treatment was indicated, except for observation.

At 3 months, the mother noticed a color change in the OD appearance in her baby and addressed an ophthalmologist, who found the above-mentioned changes (photophobia, epiphora, and spontaneous hyphema) at the clinical examination and sent the child to a tertiary center for further investigations.

**Fig. 1 a,b,c:**
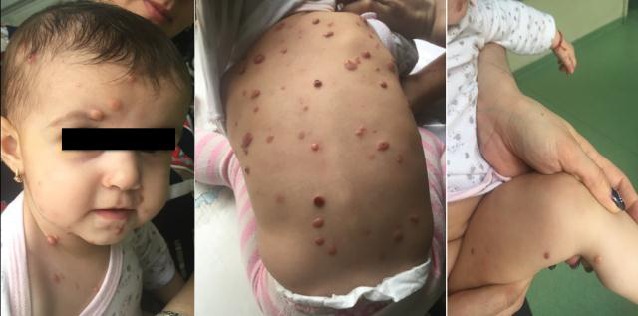
Numerous cutaneous nodules, varying in color from yellowish to bright red, mostly spread onto the head/ neck region and thorax; fewer lesions were noted on the arms and legs

The general clinical examination was completely normal, except for the numerous cutaneous nodules (**Fig. 1a,b,c**), varying in color from yellowish to bright red, mostly spread onto the head/ neck region and thorax. Fewer lesions were noted on the arms and legs. All further investigations (complementary exams, laboratory findings, and imaging scans) were within the normal limits and revealed neither lesions onto the internal organs, nor the presence of any other systemic alteration.

In the ophthalmology exam, we found visual acuity in both eyes “fixes and follows” and, under general anesthesia, IOP-OD = 25 mmHg and IOP OS =12 mm Hg. Corneal diameter was increased in OD (12 mm) and normal in OS (10 mm), aspect which coincided with increased axial lengths in OD (19.12 mm) compared to OS (18.49 mm) by “A” scan ultrasound methods.

**Fig. 2:**
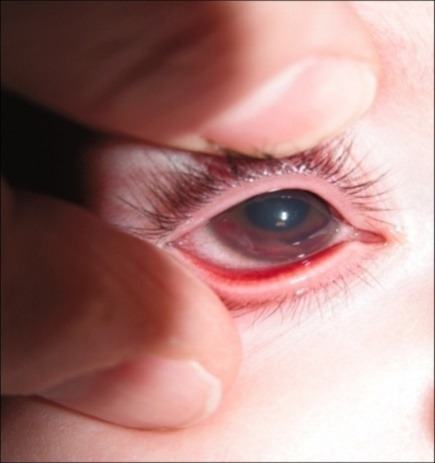
Anterior segment (OD)

**Fig. 3:**
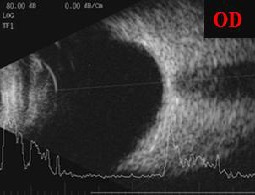
OD-B scan revealed normal structures; no JXG suggestive lesions appeared in the retina, choroid, optic nerve

Anterior segment examination found all structures with normal appearance in OS, while in OD there were visible changes such as: mild epithelial corneal edema, peripheral iris synechiae/ 360*, thick fibrinoid membrane on the surface of the iris, 2mm hyphema and a wide blood clot on the surface of the iris (11-4 o’clock meridians) – **Fig. 2**. Fundus examination could not be performed in OD due to low visibility, whereas OS aspect was normal. B scan ultrasound examination in OD detected no abnormalities in the choroid, retina, or optic nerve (**Fig. 3**).

Based on all these findings we established the diagnosis of OD - Pediatric secondary inflammatory glaucoma in the context of juvenile xanthogranuloma with ocular (iris) involvement. Short course of topical corticosteroids (dexamethasone) and close monitoring for the next 3 weeks was indicated. Since no improvement was detected within this interval and persistent hyphema, fibrinoid iris membrane (more dense in inferonasal quadrant) were present, surgical intervention became mandatory in order to avoid future and more severe complications in this child.

**Fig. 4:**
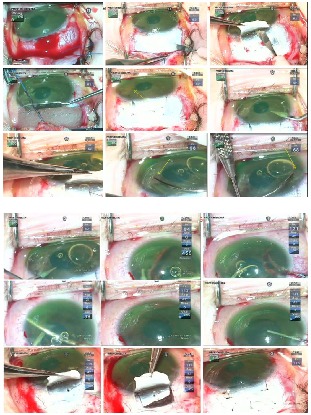
OD Augmented trabeculectomy with mitomycin C (0.02%, 3 minutes) and trabeculotomy; difficult rotation in the anterior chamber of the trabeculotomes due to the fibrinoid membrane adherent to the iris root in the AC angle; removal of the iris fibrinoid membrane, mild hemorrhage after removal, patient 12 o’clock peripheral iridectomy

**Fig. 5:**
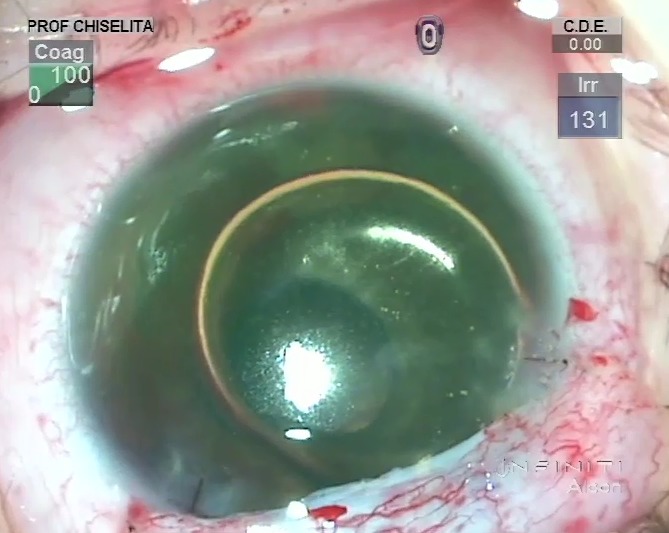
OD aspect at the end of the surgical intervention; mild hemorrhage in the AC after removal of the iris membrane

In this respect, augmented trabeculectomy with mitomycin C (0.02%, 3 minutes) and trabeculotomy were performed in November 2016. **Fig. 4** depicts the most important surgical steps. Surgery was uneventful and the outcome is presented in **Fig. 5**. 

The thick plaque extracted from the surface of the iris together with the tissue resulted from the peripheral iridectomy (at 12 o’clock) were sent to histology examination, including immunohistochemistry markers.

Early post operation medication included topical tobramycin, dexamethasone and non-steroidal anti-inflammatory drugs, 5x/ day; phenylephrine was added x3/ day to prevent recurrent bleeding in the anterior chamber. IOP was well controlled in OD (range between 8-10 mmHg) during the follow up visits. Corticosteroids were slowly tapered in the next months, until complete cessation with favorable clinical evolution.

**Fig. 6:**
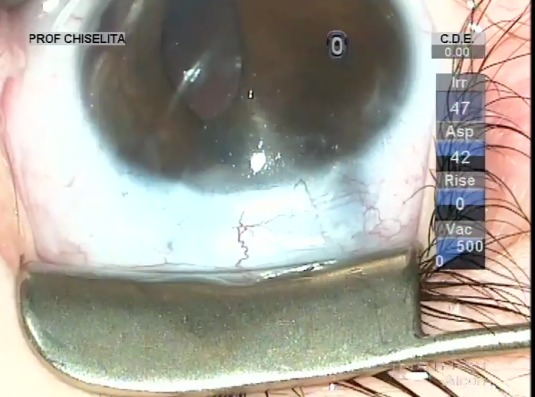
OD aspect at 5 months post operation. Thin, avascular filtering bleb, rare microcysts visible in the superior sector on the conjunctiva, diffuse sub-conjunctival filtration; clear cornea, tractioned pupil (the inferior quadrant by small SIPs); no active bleeding is visible in the AC, nor remnant/ new membranes; transparent lens

5 months after surgery, the OD was “clinically silent” (**Fig. 6**); no local recurrences were noted and the IOP was 10 mmHg without any medication. General status was stationary, since no new skin lesions on the internal organs were detected at the ancillary tests performed regularly (abdominal/ pelvic ultrasound examination, transfontanellar ultrasound and laboratory blood tests). Fundus examination was normal in both eyes; glaucoma damage was not found in OD (C/ D ratio = 0.2). 

Initial pathology examination required further investigations, as a temporary suspicion of myelomonocytic proliferation appeared in the biopsied iris tissue. A thorough pathology examination and specific IHC markers such as CD68 intensely positive in the area of active cell proliferation, factor XIIIa – intensely positive, Vimentine – mildly positive in the area of active cell proliferation, confirmed the XJG diagnosis. Langerine, CD1a, MPO, CD15, S100, HMB45, T311, MITF – were all-negative in the analyzed tissues (skin and iris) and excluded other diagnosis (Langerhans histiocytosis, myelomonocytic leukemia, etc.). Fragments of deep adipose tissue included dense cellular proliferation, without a clear demarcation zone, extending also towards the periadnexal space, dissecting from place to place collagen lamellas (deep expansive pattern growth with spindle cell morphology, scarce areas of epithelioid proliferation with medium/ large cells); frequent mitosis (21 mitosis/ 10HFP); minimal inflammatory infiltrate – lymphocytes and eosinophils. This description pleaded for a nonlipidized, mitotically active JXG, with prominent spindle cell morphology. Some details are shown in **Fig. 7a**,**b**,**c**,**d** and **Fig. 8**.

**Fig. 7a:**
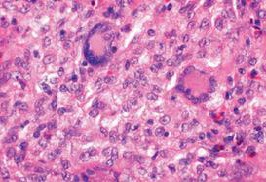
Cutaneous JXG with Touton giant cells displayed by hematoxylin-eosin (HE) stain

**Fig. 7b:**
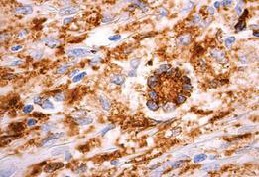
Cutaneous JXG with CD68 +++ IHC stain

**Fig. 7c:**
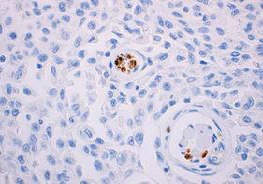
Cutaneous JXG with S-100 negative IHC stain

**Fig. 7d:**
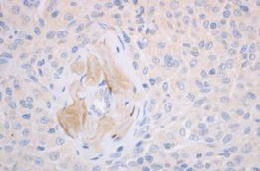
Cutaneous JXG with CD1a negative IHC stains

**Fig. 8:**
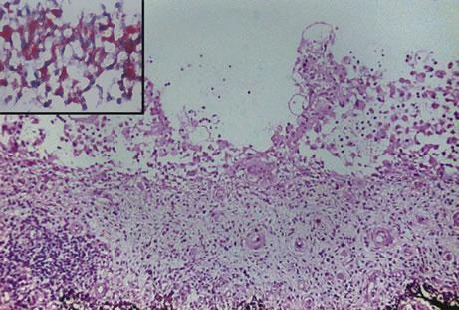
Histology exam (iris) biopsy. Hematoxylin-Eosin (HE) staining, ×25; detail, ×200). Diffuse iris infiltration of lymphocytes (monocytes); nodular aggregates on the anterior surface of the iris (histiocytes)

## Discussions

Histiocytic disorders consist mainly in two types: Langerhans cell histiocytosis (LCH) and non-Langerhans cell histiocytosis (non-LCH). JXG is a rare disorder, but it is the most common among the non-LCH forms, with benign evolution [**5**]. Lesions appear spontaneously, during the 1st year of life; as it was the case of our patient, and resolve spontaneously later in childhood. The most frequent areas involved are the head and neck region, but extracutaneous lesions are also described [**3**,**5**]. Organ involvement is very rare and happens predominantly in patients with multiple skin nodules, as the case we presented hereby [**7**]. The majority of the patients with ocular lesions are young children typically younger than 1 year, similar to the 4 months old baby, subject of this case report. Ocular lesions usually involve the iris, but have also been reported in the orbit, optic nerve, retina, and choroid [**8**,**9**]. Iridocyclitis, hyphema, and secondary glaucoma are frequent presenting signs [**10**] and represent serious complications that may cause blindness if unrecognized or left untreated [**11**]. Zimmerman identified 5 clinical patterns for intraocular involvement in infants and young children: asymptomatically localized or diffuse iris tumor, unilateral glaucoma, spontaneous hyphema, red eye with signs of uveitis and congenital/ acquired iris heterochromia. All these signs [**12**] were found in our patient. 

Microscopically, the *ocular tumor cells* are rich in cytoplasm, which is light and eosinophilic or fine granular-like. The nucleoli are round or kidney-shaped. Most of the cells are mononuclear, and some can see two or three unclear nucleoli. The number of Touton cells is decreased compared to skin JXG lesions or even missing; inflammatory cells are also rare [**13**]. This could lead to some degree of confusion for inexperienced pathologists.

Microscopy of *skin lesions* in JXG describes a diffuse invasion of numerous tissue cells accompanied by a small amount of lymphocytes and eosinophils that can be seen in the early stage of JXG. Some tissue cells have light and hollow cytoplasm, and the nucleus is small in round or ovoid shape without atypia. In some cells, the nuclear groove and an unclear nucleus can be seen, which are often absent in Touton cells. Invasion of foam cells, foreign body giant cells and Touton giant cells can be seen in mature stage. The nucleus of Touton cell is garland-like, which is the typical characteristic of juvenile xanthogranuloma. A lot of fibroblasts are visible at the late stage, and fibrosis replaces the infiltration [**13**]. Collecting tissue samples from different stages of the skin lesions might lead to different descriptions in the same patient, as it was in our case.

The differential diagnosis relies on the immunohistochemical study. JXG lesions classically stain with macrophage markers including CD68 or Ki-M1P and anti F XIIIa, vimentin and often anti-CD4. As such, JXG expresses CD68, lysozyme and FXIIIa, but not CD1a. LCH expresses S-100 protein and CD1a, but not CD68, lysozyme and FXIIIa. CD1a is a relatively specific marker for LCH and it is not expressed in non-LCH diseases [**14**]. 

In our case, multiple biopsies were collected and various interpretations were given. According to Dehner, neither factor XIIIa negativity, nor S-100 positivity should preclude the diagnosis of JXG [**15**]. Therefore, the first attempt to characterize the skin biopsy fragments was made, a diagnosis of Lanherhans Cells Histiocytosis was discussed, based on CD68+++, CD 45+, MPO+, CD1a-, S-100-, desmin -, MY14-. Further IHC exams stated that elements such as positive markers for histiocytes and monocytes (CD68+++ and CD14+), Langerine- and CD1a – could exclude HCL or another tumor with dendritic undetermined cells, that are usually Langerine -, but CD1a+. Spindle cell morphology, corroborated with fascein+, FXIIIa- could exclude a myelomonocytic proliferation. Third and most comprehensive IHC evaluation showed positive markers such as CD 68 (+++), factor XIIIa(++), Vimentine (+), whereas Langerine, CD1a, MPO, CD15, S-100, HMB45, T311, MITF were all negative. All the above-mentioned IHC markers are consistent with JXG characteristics.

In most cases with JXG, S-100 protein is non reactive, but scattered cells may show weak cytoplasmic reactivity, unlike the more diffuse and intense reaction of Langerhans cells [**16**]. Probably this was the reason for the initial confusion that was made with malignancies that required a bone marrow biopsy in this child at 3-4 weeks after birth.

JXG lesions beyond skin have different histological characteristics with cutaneous JXG, and are easy to be confused with Langerhans cell histiocytosis (LCH) [**17**], therefore the confusion in this case, when an iris fragment was analyzed; yet, JXG and LCH might be found simultaneous in the same patient [**18**]. A “triple disease” (18% of the cases) defined as JXG plus neurofibromatosis type 1 (NF-1) and juvenile chronic myelogenous leukemia (JCML) has been reported, but it was not confirmed in our patient.

From the three characteristic histologic patterns of JXG: early JXG (EJXG), classic JXG (CJXG), and transitional JXG (TJXG) [**19**,**20**], we found elements consistent for all forms in our patient: CJXG due to abundant vacuolated, foamy histiocytes with Touton giant cells, TJXG due to predominance of spindle-shaped cells resembling benign fibrous histiocytoma with foamy histiocytes and EJXG forms due to cells with little lipid and relatively more mitoses than the others. The high number of mitosis found in our case of non-lipidized JXG could be explained by the immature or evolving character of this histologic subtype.

Regarding the therapeutic options, patients with a single or only a few lesions need no therapy; excisional biopsy can be considered, but only for cosmetic reasons. Due to potential blinding complications, treatment of iris JXG should be promptly considered. Usually, corticosteroids are the main stay of treatment, but refractory cases have been described, therefore an escalation of treatment needs to be approached, if the patient’s status deteriorates and the disease progresses despite conventional treatment. Surgery serves both diagnosis and treatment, since it provides a tissue specimen and excisional biopsy is curative, since the lesions once excised do not recur [**21**]. Refractory cases or for the patients who have systemic disease require radiotherapy (low dose) or chemotherapy similar to LCH protocols [**22**]. Glaucoma surgery (trabeculectomy and trabeculotomy) was “curative” for the iris JXG in this patient, and prevented complications of intraocular inflammation and increased IOP. Postoperatory management included only topical corticosteroids and NSAID with positive outcome during the follow up period. We recorded no recurrence, either local or systemic in this patient, but still, a close monitoring is needed in this child.

## Conclusions

We presented a difficult case of a mitotically active JXG in iris and skin that requested multidisciplinary approach, involving an ophthalmologist, pathologist, pediatrician, dermatologist, etc. After treating the ocular complication, the situation was declared temporarily stable, but the long-term prognosis is yet guarded and further monitoring is needed.

Although JXG is described as a benign tumor, some histological aspects might be confounders for inexperienced pathologists; therefore, the definitive diagnosis should be made only by IHC markers just to avoid errors.

As general recommendation we advice the ophthalmology fellows to consider the JXG diagnosis in every case of spontaneous hyphema they find in infants and young children, since spontaneous hyphema is the most common presentation in ocular JXG in this range of age. Treatment should be promptly considered to avoid potential blinding complications. 
